# The Matron's Books

**Published:** 1907-07-20

**Authors:** 


### Nursing Administration.

### THE MATRON'S BOOKS.

### II.—PROBATIONERS' PROGRESS BOOK.

In a previous article some specimens were given of registers containing particulars of the probationers' training. Each method has its own advantages, and the following ruling attempts to combine the best features of all.

This may conveniently find a place on the page facing the above in the register.

In addition to these records the certificate issued to the nurse is copied into a separate book which contains particulars of all certificates, and may be in the form of counterfoils to the certificates themselves.

Probationer's Register.

*Name*...................... *Appointed*.................. ... *Folio*..................

| Ward | Nature of Work | Dates From | Dates To | No. of Days | No. of Nights | Special Cases | Report of Sister | Off Duty Illness, or Leav |
|---|---|---|---|---|---|---|---|---|
| B ... | Male Surgical ... | Sept. 1 | Sept. 30 | 30 | | | Painstaking, but slow. Signed A. B. | |
| | Off duty ... ... | Oct. 1 | Oct. 14 | 14 | ...... | ...... | ... ... | Holiday |
| H ... | Gynæcological ... | Oct. 14 | Nov. 28 | | 45 | | Trustworthy, good memory. Signed C. D. | |

At the bottom of the page a *résumé* should be given.

| Name ......................................... | Days | Nights |
|---|---|---|
| Total of surgical work ... ... ... | | |
| Total of medical work ... ... ... | | |
| Illness ... ... ... ... ... | | |
| Holidays, leave, and off duty ... ... | | |

Thus the whole career of the nurse is easily ascertainable at a glance, with the judgment pronounced on her work by successive superiors. Unless this summary is performed strange mistakes are liable to occur, and that they do occur and result in curiously one-sided training many nurses whose skill was never properly cultivated during their pupil years can testify. In general there is more danger of laxity in keeping the Registers of Probationers in small than in large hospitals. Even the Cottage Hospital with but half a dozen beds is in duty bound to keep an exact account of the opportunities afforded to the probationer, and the proportions of day and night duty which fall to her. Such a description of the training would do much to reconcile the matron of a general hospital to a candidate having been already under instruction, for it would show her that the previous experience had not been gained in that atmosphere of muddle and rush which it is to be feared too often pervades the cottage hospital posing as training school. The number of classes and lectures attended has next to be registered, with the result of all examinations.

| Subjects | Number Attended: Lectures | Number Attended: Classes | Result of Examinations: Marks: Max. | Result of Examinations: Marks: No. Obtained | Result of Examinations: Examiner's Report |
|---|---|---|---|---|---|
| Physiology ... | | | | | |
| Anatomy ... | | | | | |
| Hygiene ... | | | | | |
| Theoretical nursing | | | | | |
| Practical nursing | | | | | |
| Cookery ... ... | | | | | |
| Bandaging, etc. ... | | | | | |

The value of keeping these minute records of the probationer's doings has been already emphasised. It is not sufficient to keep a straggling account of the wards in which the time has been spent. The *résumé* which shows the total amount of experience, night and day, afforded to the nurse in each department, is of the utmost value.

The register may, under able management, supply in small hospitals the place which is filled in large institutions by emulation. It serves to make every-day events matters of importance, and it is the feeling that things do not much matter which is the great drawback to the small training schools. There may, in a non-clinical provincial hospital, be even greater opportunities of learning the work than in an important training centre, and ample material for instruction, but unless the nurses can be brought to understand the value of working up to the top of their abilities all the time, they will not become smart in the performance of their duties. A thoroughly well kept register, kept with the full knowledge of the nurses, is the greatest possible assistance in helping the probationers to realise that every day's work counts in the sum of their training.

We are disposed to think that much good would result if each probationer were encouraged to keep a record similar in character to the above wherein every change in her work, every opportunity for gaining fresh experience were entered by herself. Doubtless there may be advantages in keeping the reports received from the various sisters relative to the probationer's work confidential. Such reports might degenerate into commonplaces were they presented with the knowledge that they would be communicated to those they concern. But on the other hand a keen stimulus would be supplied were the probationer made aware at the conclusion of her three months' work exactly what judgment had been passed on her, and the strongest motive would be established for maintaining a high level in this unerring record of training days—a record which would in years to come be regarded as one of her best treasures.

